# A case of tension faecopneumothorax after delayed diagnosis of traumatic diaphragmatic hernia

**DOI:** 10.1186/s40792-018-0447-y

**Published:** 2018-04-20

**Authors:** Tien Yew Chern, Allan Kwok, Soni Putnis

**Affiliations:** Department of Surgery, Wollongong Hospital, Locked Bag 8808, South Coast Mail Centre, NSW 2521 Australia

**Keywords:** Diaphragm, Diaphragmatic injury, Diaphragmatic trauma, Penetrating trauma, Tension pneumothorax, Faecopneumothorax, Stercopneumothorax

## Abstract

**Background:**

Traumatic diaphragmatic injuries from blunt or penetrating trauma are difficult to detect in the acute setting and, if missed, can result in significant morbidity and mortality in the future. We present a case demonstrating the natural progression of this resulting in faecopneumothorax, which is a rare but serious presentation.

**Case presentation:**

A 22-year-old young man presented with left upper quadrant and chest pain, nausea, vomiting, and intermittent obstipation with a background of previous lower chest wall stabbings. Computed tomography demonstrated a diaphragmatic hernia containing the splenic flexure of the colon, but he declined treatment and self-discharged. He presented three more times with similar symptoms and self-discharged within a 2-week period and finally presented dyspnoeic and septic. Computed tomography demonstrated tension faecopneumothorax from the perforated colon. He was taken to theatres and found to have a 3-mm perforation at his splenic flexure and underwent a segmental resection of the affected colon, intrathoracic washout, and biological mesh repair of his diaphragmatic hernia. He remained alive and postoperative recovery was uneventful.

**Conclusions:**

A review of the literature demonstrates the rarity of traumatic diaphragmatic injuries resulting in faecopneumothorax with only a few case reports in the last 50 years. We present a case demonstrating a natural progression of the condition and highlight the importance of having a high index of suspicion of diaphragmatic injuries in the trauma setting.

## Background

Faecopneumothorax secondary to traumatic diaphragmatic injury (TDI) is a rare but serious presentation. To date, there have only been 12 case reports in the literature in the past 50 years [1–12]. The difficulty in diagnosing or ruling out traumatic diaphragmatic injuries from penetrating trauma can result in missed injuries and delayed diagnoses leading to significant morbidity and mortality. We present a case of tension faecopneumothorax resulting from a delayed diagnosis of diaphragmatic hernia containing colon resulting from stab wounds 2 years prior.

## Case presentation

A 22-year-old young man presented to the Emergency Department at our hospital with severe left upper quadrant and chest pain, nausea, vomiting, and intermittent obstipation for the past week. He had a history of substance abuse and mental health issues and 2 years ago was stabbed multiple times following an altercation including two in the left lower chest with associated haemopneumothorax requiring exploration and washout. Without follow up, he presented three times to a country hospital and discharged against medical advice every time in the 2 weeks prior to his final presentation at our hospital. The first of these three presentations was triggered by severe left upper quadrant pain and subsequent computed tomography (CT) demonstrated a delayed left diaphragmatic hernia containing the splenic flexure of the colon for which he declined treatment. The next presentation occurred 2 days later when he developed obstipation and was transferred from the country hospital to our hospital but again self-discharged against medical advice while awaiting surgery. The third presentation occurred the day prior to his final presentation but again self-discharged prior to repeat imaging.

**Fig. 1:**
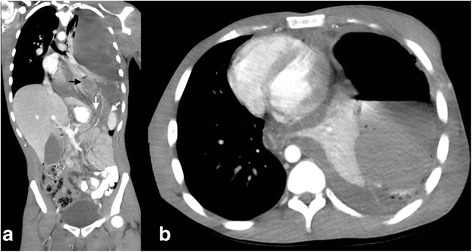
Computed tomography. **a** Coronal view demonstrating hernia of diaphragm containing large bowel and a complex hydropneumothorax of the left thoracic cavity consistent with perforation of the herniated bowel causing faecopneumothorax. Black arrow points to a loop of large bowel within the thoracic cavity, and white arrows point to the diaphragmatic hernia defect. **b** Axial view demonstrating collapse of the left lung due to the complex hydropneumothorax and contralateral mediastinal shift

On his final presentation at our hospital, he was febrile to 38.9 °C and tachycardic to 140 bpm at triage but remained normotensive and was saturating normally. His abdomen was non-distended but tender and guarded in the epigastrium. White cell count was 18.74 × 10^9^/L, CRP was 89 mg/L, venous lactate was 1.5 mmol/L, and electrolytes were generally deranged (see Table 1). An urgent repeat CT scan of his chest and abdomen demonstrated a large left hydropneumothorax with mediastinal shift to the right (Fig. 1). Following the scan, he became tachypnoeic with evidence of a tension pneumothorax. A chest drain was inserted and foul-smelling gas with large amounts of bowel contents was drained. The patient was transferred to theatres.

**Table 1:** Blood results on the day of the patient’s final presentation

Blood results	
Sodium	125 mmol/L (elevated)
Potassium	7.0 mmol/L (elevated)
Chloride	84 mmol/L (reduced)
Bicarbonate	16 mmol/L (reduced)
Creatinine	70umol/L
White cell count	18.74 × 10^9^/L (elevated)
Neutrophils	18.14 × 10^9^/L (elevated)
C-reactive protein	89 mg/L (elevated)
Haemoglobin	158 g/L
Venous lactate	1.5 mmol/L

**Fig. 2:**
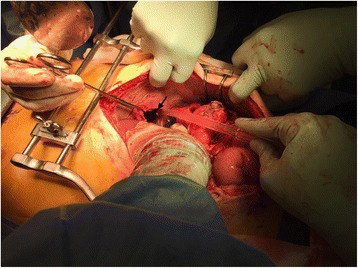
Intraoperative view of the inferior aspect of the left diaphragm. Black arrow points to the diaphragmatic hernial defect

**Fig. 3:**
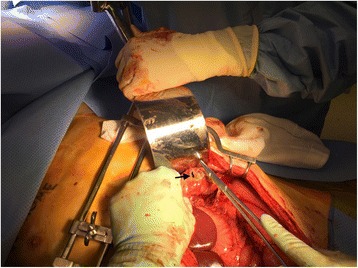
Intraoperative view of the reduced splenic flexure. Black arrow points to the colonic perforation

Laparotomy demonstrated a 7–10-cm left diaphragmatic hernia (Fig. 2) containing the splenic flexure of the colon with a 3-mm perforation (Fig. 3) causing gross left intrathoracic faecal contamination but no intra-abdominal contamination. The descending colon was mobilised, and a segmental resection of the splenic flexure was performed with primary stapled anastomosis. The left pleural cavity was extensively washout out, two chest drains were placed, and the diaphragmatic defect was closed with an on-lay biosynthetic mesh also placed and secured. A partial splenic infarct was noted at surgery likely due to hilar vessels being included in the hernia. This was conservatively managed. Post-operative recovery was uneventful.

## Discussion

TDI is a rare injury occurring in 0.8 to 8% of all traumas and has thus been difficult to study, with most being retrospective single-centre trials over multiple years with associated inherent bias [13, 14].

TDIs can be arbitrarily divided by anatomy into left and right diaphragmatic injuries with right-sided injuries being associated with increased morbidity and mortality [15]. While post-mortem studies show relatively equal incidence, recent studies from patients presenting to trauma centres, left-sided injuries (50 to 76.1%) seem to be more common than right-sided injuries (23.9 to 47%) [13, 16, 17].

TDIs can also be classified by mechanism, by either blunt injuries or penetrating injuries with traditionally blunt injuries bearing more of the burden (75%) than penetrating injuries (25%) [18, 19], although a few recent studies have demonstrated the opposite, although this may be more related to geography [14, 16].

TDIs from blunt trauma occur in high-energy events causing a sudden rise in intra-abdominal pressure overcoming the strength of diaphragm thus rupturing it. These generally occur in high-energy motor vehicle accidents and falls and are associated with a higher mortality due to the severity of other associated injuries in high-impact events. TDIs from penetrating injuries can generally occur from knife to gunshot injuries. As the diaphragm’s location can vary depending on respiration, any penetrating injury from spinal levels T4 to T12 may risk diaphragmatic injury. Diaphragmatic injuries per se can cause significant morbidity and mortality through loss of adequate function of the diaphragm or herniation of contents in the acute setting. More importantly, the injuries associated with the severity of an event that can cause diaphragmatic have significant effects on mortality [20]. Hanna et al. demonstrated that TDIs associated with traumatic brain injury and haemorrhagic shock increased the risk of death and separately significant injuries in blunt injuries which include head trauma and rib fractures and those in penetrating injuries which include visceral injury and age [16].

TDIs can only be completely ruled out by direct vision and can be demonstrated operatively in a trauma laparotomy or laparoscopy with a sensitivity of 100% and specificity of 87.5% with the latter [21]. However, if there is no indication for that in the first place, the diagnosis will have to depend on imaging including chest x-rays, ultrasound, magnetic resonance imaging, or computed tomography (CT). The latter is increasingly used and studied, and while sensitivity has traditionally been considered low [19, 21], a recent study has demonstrated greater sensitivity (82%) and specificity (88%) [22]. Because penetrating injuries tend to cause smaller diaphragmatic defects, even CTs may not be able to detect these injuries [16]. Therefore, it has been traditionally recommended that laparoscopy be performed in patients with high clinical suspicion for injury, which can also facilitate a laparoscopic repair. However, this may lead to a high negative laparoscopy rate of 75% reported by one study, which would be unacceptable in most settings [23]. Considering this, a recent randomised controlled trial in South Africa has demonstrated the safety of observation and follow-up including imaging over a mean of 24 months for left-sided stab wounds [24]. The relevance of this study in other countries such as Australia is difficult given the difficulty in following up following stabbing who tend to be young, mobile, and unwilling to present for routine follow-up. Therefore, most studies have either not included follow-up data or have included little data. Hanna et al., for example, have only been able to follow-up with 17.1% of their subjects [16].

The treatment of TDIs includes simple or continuous closure with typically non-absorbable suture which can be performed laparoscopically or open. Depending on the size of the defect, mesh may have to be used but given the high chance of contamination, biosynthetic mesh may be used [16, 20, 25]. Complications include recurrence of hernia, but the literature is scarce [26] with the only study being Hanna et al. [16] reporting 2 cases of 13 patients followed up.

An early description of TDIs by Grimes [27] suggested three phases of rupture. The acute phase refers to the initial presentation; the delayed phase refers to transient herniation and hence a period absent of symptoms and lastly the obstruction phase where hernias have become long-standing and can become complicated.

Due to the difficulty of initial diagnosis and often the absence of symptoms, occult injuries do occur in 12–66% [28, 29] and can present any time from 2 months up to 50 years [30, 31]. Despite Grime’s early descriptions, due to the overall lack of numbers, the natural history of an occult TDI is not well known, although some, especially on the left, result in diaphragmatic hernias, eventually becoming symptomatic and can cause much morbidity and mortality [32]. It is thought that respiration gradually increases the size of the defect through radial pulling of the surrounding muscle fibres during inspiration, thus allowing viscera to herniate through what was initially a small defect [23]. That and the negative pressures of the chest encourage herniation of organs such as the stomach, spleen, and small and large bowel. While initially asymptomatic, patients can later present with symptoms such as chest or abdominal pain, dyspnoea, or symptoms of bowel obstruction. Eventually if left untreated, some of these hollow viscus organs perforate causing severe sepsis.

Some of these have resulted in faecopneumothorax such as our patient who has had an occult TDI resulting in diaphragmatic herniation of the colon. The CT 2 years prior had not shown the diaphragmatic defect and expert review of the old images had also failed to identify the defect, further demonstrating the difficulty of the diagnosis. While the sites of the wounds were certainly suspicious, there was no indication for a laparoscopy at the time. When he became symptomatic and there was a clear indication for repair, he discharged against medical advice several times frustrating attempts at definitive treatment. Because he still had capacity, his wishes had to be respected. It was only when emergency life-saving treatment was required that he finally gave consent. This highlights the difficulty in providing early appropriate treatment and that while follow-up might have been ideal, it was unlikely that this patient would have done so.

## Conclusion

The diagnosis of TDIs from penetrating injuries is difficult, and if missed, a diaphragmatic hernia could result, which might eventually cause faecopneumothorax, a severe complication that could result in death. Therefore, a high index of suspicion for TDIs in trauma is required, and if clinically suspicious, laparoscopy should be performed with diaphragmatic inspection.

## Abbreviations

BPM: Beats per minute
CT: Computed tomography
TDI: Traumatic diaphragmatic injury
